# Anesthetic Implications for Cesarean Section in a Parturient with Complex Congenital Cyanotic Heart Disease

**DOI:** 10.1155/2018/2616390

**Published:** 2018-03-29

**Authors:** Huili Lim, Chuen Jye Yeoh, Jerry Tan, Harikrishnan Kothandan, May U. S. Mok

**Affiliations:** Department of Anesthesia and Intensive Care, Singapore General Hospital, Singapore

## Abstract

The discordance between increased physiological demand during pregnancy and congenital cardiac pathology of a parturient is a perilous threat to the maternal-fetal well-being. Early involvement of a multidisciplinary team is essential in improving peripartum morbidity and mortality. Designing the most appropriate anesthetic care will require a concerted effort, with inputs from the obstetricians, obstetric and cardiac anesthesiologists, cardiologists, neonatologists, and cardiothoracic surgeons. We report the multidisciplinary peripartum care and anesthetic management for cesarean section (CS) of a 28-year-old primigravida who has partially corrected transposition of the great arteries, atrial and ventricular septal defect, dextrocardia, right ventricle hypoplasia, and tricuspid atresia.

## 1. Introduction

In Asia, congenital heart disease occurs in approximately 9.3 per 1000 live births [1]. Modern medicine has substantially improved the survival of patients with major congenital heart disease and extended their life expectancy beyond reproductive age. The complex nature of the cardiac anomaly coupled with the wide array of sophisticated temporizing or corrective procedures performed on these patients translates into a daunting task for the anesthesiologists. The physiological changes associated with pregnancy and delivery compound the problem further.

Written consent was obtained from the patient for publication with approval from local ethics committee. This manuscript adheres to the applicable EQUATOR guidelines.

## 2. Case Description

**Figure 1:**
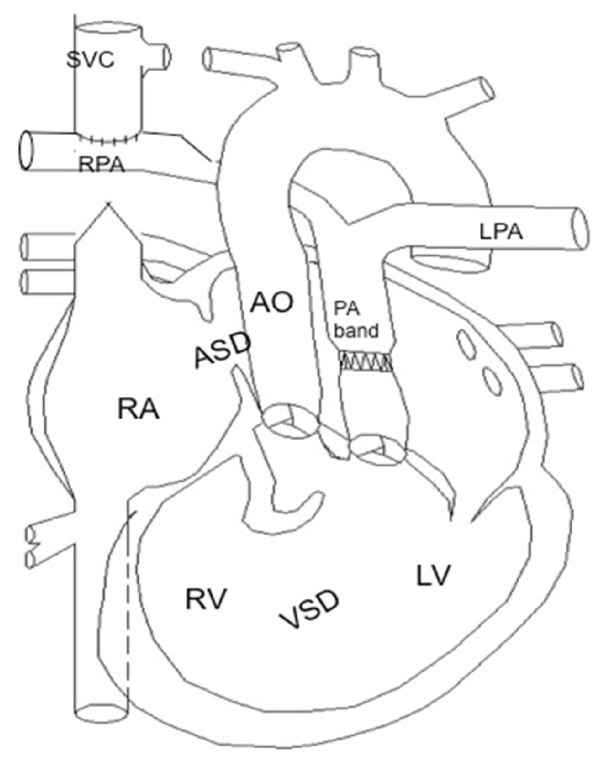
*Patient's abnormal cardiac anatomy (picture courtesy of cardiologist Dr. Tan Ju Le)*.* (1) Transposition of great arteries*: the morphologic left ventricle is connected to the pulmonary artery (PA) and pulmonary circulation; the hypoplastic right ventricle is connected to the aorta and systemic circulation.* (2)* The* tight pulmonary artery band* limits blood flow from the left ventricle into the pulmonary circulation to prevent development of pulmonary hypertension and to direct oxygenated blood from left ventricle to right ventricle through the large nonrestrictive* ventricular septal defect (VSD)* and then from right ventricle to systemic circulation.* (3)* Systemic venous blood from the superior vena cava enters the pulmonary circulation for oxygenation via the* bidirectional cavopulmonary connection*.* (4)* Systemic venous blood from the inferior vena cava returns to the right atrium and enters left atrium via the large nonrestrictive* atrial septal defect (ASD)*, from left atrium to the morphologic left ventricle. Due to the congenital* tricuspid atresia*, there is no blood flow form right atrium to right ventricle.* (5)* Oxygenated blood from the lungs enters the pulmonary veins, flows to left atrium and left ventricle and then through the ventricular septal defect to right ventricle. Both the hypoplastic right ventricle and the left ventricle (via the ventricular septal defect) eject blood into the aorta. Bidirectional shunting occurs across the ventricular septal defect with mixing of oxygenated and deoxygenated blood.

Our patient is a 28-year-old primigravida who presented with New York Heart Association (NYHA) class III symptoms at 17 weeks' gestation. She was diagnosed with complex congenital cyanotic heart disease at birth and her cardiac malformations included transposition of great arteries, large nonrestrictive ventricular septal defect (VSD) and atrial septal defect (ASD), dextrocardia with atrial and abdominal situs solitus, hypoplastic right ventricle, tricuspid atresia, and multiple aortopulmonary collaterals to the right lung. She had pulmonary artery banding at 6 weeks of age and bidirectional cavopulmonary connections (also known as Glenn's shunt) were performed at the age of 18 (see Figure 1.) Completion of Fontan's procedure was held off indefinitely because of an incidental finding of cerebral arteriovenous malformation (AVM). The AVM was deemed unsuitable for embolization due to its connection with a large venous pouch. She had been counselled against pregnancy by her cardiologist before conception. The patient's other comorbidities include mild scoliosis, prolapsed intervertebral disc between the fourth and fifth lumbar vertebrae, previous infective endocarditis complicated by a cerebrovascular accident (full neurological recovery from left-sided numbness), and compensatory polycythemia (hemoglobin concentration: 20–22 g/dL).

At 17 weeks' gestation, the patient was admitted by her cardiologist because of increasing cyanosis and dyspnea. Her oxygen saturation (SpO_2_) on air decreased from 90% before pregnancy to 80% on admission. Obstetrical work-up revealed a healthy viable fetus. Serial transthoracic echocardiography showed an ejection fraction of 55% with bidirectional shunt across the VSD. Due to pulmonary artery banding, transpulmonary gradient was 41 mmHg. The obstetric anesthesiologist and obstetrician were alerted from the time of admission. The patient was again counselled extensively regarding the risks of further maternal decompensation and fetal anomaly. However, she remained keen to continue with the pregnancy. The patient stayed in the cardiology high dependency ward for bed rest, oxygen therapy, and monitoring by a cardiologist who was specialized in adult congenital heart disease from 17 weeks' gestation until the point of delivery.

A multidisciplinary team comprising cardiologists, obstetricians, obstetric and cardiothoracic anesthesiologists, cardiothoracic surgeons, and neonatologists was established from the time of admission. The discussions were led jointly by the obstetrician and the anesthesiologists. The team met regularly with the following agenda: (1) update on patient's condition, (2) update on fetal condition (after 24 weeks), (3) plans for an early elective delivery, and (4) formulation of an emergency plan in the event of acute maternal decompensation. The multidisciplinary team had consensus that maternal condition would deteriorate in the 3rd trimester, and cesarean section (CS) should happen before 28 weeks of gestation to avoid maternal decompensation. Nearing the end of 2nd trimester, the patient was demonstrating NYHA class IV symptoms and would desaturate during minimal efforts such as eating and speaking. Her highest SpO_2_ value prior to delivery was 85% on a nonrebreathing mask with 15 L/min of oxygen. A decision was made for an elective CS to be performed at 27 weeks of gestation in view of her worsening functional capacity. This was to allow the conduct of anesthesia and surgery under controlled conditions.

A general anesthetic technique was jointly agreed upon by the cardiac and obstetric anesthesiologists because of the potential for less hemodynamic instability. Members of the multidisciplinary team were present in the operating theatre on the day of surgery. The perfusionists were also on standby in preparation for the possible need for extracorporeal membrane oxygenation (ECMO).

Equipment necessary for both maternal and neonatal care and monitoring were available, including transesophageal echocardiography (TEE), nitric oxide for inhalation, and neonatal resuscitator (medical device). Intravenous cardiovascular drugs (nitroglycerine, norepinephrine, epinephrine, and milrinone) were prepared. Before the induction of anesthesia, the vascular access sites for emergency ECMO support were identified and marked. Standard monitors were attached, and the left radial artery and left internal jugular vein were cannulated for intra-arterial and central venous pressure lines. The left internal jugular vein was cannulated as the right internal jugular vein was noted to be small on ultrasound imaging. A pulmonary artery catheter was not considered in this patient as it would be technically difficult. This was because the superior vena cava was anastomosed to the right pulmonary artery. Typical pulmonary artery tracing would not be obtainable as the blood flow to the lung is passive in nature due to her altered anatomy.

The patient's airway was locally anesthetized with 5 mL of 1% nebulized lidocaine and cophenylcaine sprays to attenuate the sympathetic response to laryngoscopy and endotracheal intubation. Before induction of anesthesia, an antibiotic was prophylactically administered as per institutional protocol. The surgical site was cleansed with antiseptic solution, and surgical drapes were attached. Rapid sequence induction of anesthesia was performed with cricoid pressure. Induction drugs used include fentanyl 100 mcg, ketamine 25 mg, etomidate 10 mg, and rocuronium at 1 mg/kg (research papers have supported the use of 1 mg/kg for rapid sequence induction). The trachea was intubated with a 7.5 mm endotracheal tube.

Intraoperatively, the patient was hemodynamically stable with mean arterial pressure (MAP) remaining more than 70 mmHg throughout. The SpO_2_ value remained within 10% of her baseline at 81–87%. Excessive airway pressure was avoided by setting a low tidal volume (350 mL) and a higher respiratory rate (15) for volume-controlled mechanical ventilation. Peak end expiratory pressure was set at zero. Peak airway pressure was consistently at or below 20 cm H_2_O. The patient was closely monitored for hypercapnia and hypoxia. Hypothermia was prevented with the use of Bair hugger warming blanket and Hotline fluid warmer. Preload was optimized by trending the central venous pressure (CVP readings) and was guided by real-time TEE imaging. The CVP readings were, in fact, pulmonary artery pressure readings as the superior vena cava in this patient was anastomosed to the right pulmonary artery. The hemoglobin concentration was kept above 16 g/dL (patient's baseline was approximately 20–22 g/dL). Blood products were available on standby. Air bubbles in intravenous drip sets were carefully avoided in view of the presence of VSD. Cardiac function was continuously monitored by TEE performed by the cardiologist in direct contact with the anesthesiologist.

Following the delivery of the baby, three intravenous boluses of oxytocin 1 U were administered, which achieved satisfactory uterine contraction. There were no noticeable changes in the vital signs during autotransfusion.

At the end of surgery and before tracheal extubation, bilateral transversus abdominis plane blocks were performed. The muscle relaxant was reversed with sugammadex 2 mg/kg. The hemodynamic stimulation during tracheal extubation was blunted by titrated boluses of esmolol.

After tracheal extubation, she was monitored in the intensive care unit for 1 day. She was subsequently transferred to a high dependency ward under the care of the cardiologist and had no episodes of acute desaturation during her stay. She was discharged home on postoperative day 10.

The baby was delivered weighing 870 g and was sent to neonatal intensive care unit. He was subsequently discharged well.

## 3. Discussion

In industrialized countries, maternal congenital heart disease is the most common cause of mortality in parturients with preexisting cardiovascular disease. Mortality risk of up to 30% is associated with poor NYHA class status, severe ventricular dysfunction, severe aortic stenosis, Marfan's syndrome with aortic valve lesion or aortic dilatation, or pulmonary hypertension [2].

A best evidence topic presented by Asfour et al. in 2013 deems vaginal delivery to be safer in patients who are NYHA classes I and II in labour, but an expedited instrument-assisted vaginal delivery in patients with poorer NYHA status is feasible with good analgesia. Right ventricular failure, pulmonary regurgitation, and pulmonary hypertension impart the greatest risk to both mother and baby [3]. Due to the poor maternal state of our patient antenatally and the prematurity of the fetus, the multidisciplinary team decided that CS under controlled conditions is the safest option.

After multidisciplinary discussions, we decided to perform the cesarean section under general anesthesia. We considered general anesthesia as the method of choice because of a possibly lower risk of hemodynamic instability compared to neuraxial anesthesia. Spinal and to a lesser extent epidural anesthesia will lead to profound sympathetic block. General anesthesia using carefully selected anesthetic agents known to produce less hemodynamic effects will offer more predictability and stability. In addition, general anesthesia will enable the use of TEE intraoperatively. The underlying lumbar scoliosis and disc prolapse might have complicated the administration of neuraxial anesthesia. Accidental or deliberate dural puncture occurring during attempted epidural or spinal anesthesia, respectively, might have caused rupture of the cerebral AVM secondary to a change in transmural pressure [4]. The patient was also too breathless to tolerate the supine position required for the surgery.

For patients with Glenn shunt in situ, the superior vena cava is anastomosed to the right pulmonary artery, such that blood bypasses the malformed chambers of the right heart and is shunted directly into the lungs for oxygenation. Anesthetic goals include (a) maintenance of adequate preload, (b) preservation of sinus rhythm and myocardial contractility, (c) maintenance of a low pulmonary and normal systemic vascular resistance, (d) avoidance of hypoxia, hypercarbia, acidosis, and hypothermia, which may increase right to left shunt, (e) avoidance of air bubbles in intravenous drip sets, (f) avoidance of excessive airway pressure by maintaining low tidal volume ventilation and avoiding the use of peak end expiratory pressure, and (g) maintenance of hemoglobin level above 16 g/dL. The stress response to intubation as well as surgery should be well blunted.

Central venous pressure (CVP) line was inserted in the left internal jugular vein. CVP in this patient denotes the pulmonary artery (PA) pressure as the superior vena cava is directly connected to the right PA. In addition, a central venous line is necessary for administration of inotropic drugs if required.

General anesthesia is not without its perils. A reduction in venous return due to vasodilation, myocardial depression due to anesthetic agents, and reduction in systemic vascular resistance will result in worsening of the right to left shunt in this patient [5]. Atelectasis following general anesthesia may also worsen the right to left shunt. Sympathetic discharge during laryngoscopy which is known to be more difficult in parturients [6] can lead to rupture of the AVM with devastating consequences.

Etomidate and ketamine were used as induction agents in view of their cardiovascular stability. Etomidate causes minimal changes in systemic and pulmonary vascular resistance and the cardiac output. Specifically, ketamine administration in patients with congenital heart disease has been shown to produce minimal changes in shunt direction or systemic oxygenation [7] and improve myocardial contractility [8]. Etomidate and ketamine are safe to use in obstetric anesthesia with minimal changes to uterine blood flow and no adverse neonatal outcomes [5].

After delivery of the baby, autotransfusion of blood from the uteroplacental bed back into the maternal circulation together with blood loss can increase or decrease preload causing unpredictable effects on right heart function and degree of right to left shunt. For this reason, glyceryl trinitrate, vasoconstrictors, and inotropes were made available intraoperatively to counteract these effects during surgery.

This complicated patient was under the care of the multidisciplinary team during her stay in the hospital. Without such conjoint effort, a desirable outcome would not have been possible in this complicated cardiac patient.

In conclusion, a multidisciplinary team involving obstetricians, adult congenital cardiologists, anesthesiologists, cardiothoracic surgeons, and neonatologists should be initiated at the earliest opportunity during pregnancy. Patients with partially or fully corrected congenital heart disease should also be managed in centres experienced in the care of these conditions. In the multidisciplinary team meetings, patient-specific concerns should be discussed. Detailed plans for delivery should be mapped out, and a contingency plan should be formulated in anticipation of maternal deterioration requiring emergency surgery.

The mode of anesthesia and delivery should be decided based on patient-specific factors, and the decision should be made in conjunction with the obstetrician, cardiologist, and neonatologist.
